# Muscle Strength and Glycaemic Control among Patients with Type 2 Diabetes

**DOI:** 10.3390/nu12030771

**Published:** 2020-03-14

**Authors:** Hiba Bawadi, Dana Alkhatib, Haya Abu-Hijleh, Joud Alalwani, Lina Majed, Zumin Shi

**Affiliations:** 1 Human Nutrition Department, College of Health Sciences, QU-Health, Qatar University, Doha 2713, Qatar; DA1602675@qu.edu.qa (D.A.); ha1601254@qu.edu.qa (H.A.-H.); ja1602310@qu.edu.qa (J.A.); Zumin@qu.edu.qa (Z.S.); 2 Sport Science Program, College of Arts and Sciences, Qatar University, Doha 2713, Qatar; lina.majed@qu.edu.qa

**Keywords:** muscle strength, diabetes, HbA1c, handgrip

## Abstract

Poor glycaemic control is associated with chronic life-threatening complications. This cross-sectional study examined whether there is an association between handgrip strength and glycaemic control among patients with diabetes. Data on 1058 participants aged 40 and older were collected from the National Health and Nutritional Examination Survey (NHANES). Muscle strength was assessed using a handgrip dynamometer, and glycaemic control was assessed using HbA1c. Handgrip strength was presented as age- and gender-specific quartiles, with participants in quartile 1 having the lowest handgrip strength and participants in quartile 4 having the highest handgrip strength. Logistic regression analyses were used to assess the association between handgrip strength and poor glycaemic control among participants with diabetes. Three models, each adjusted to include different variables, were employed. Odds ratio (OR) values revealed no association between handgrip strength and glycaemic control after adjusting for age, gender, and race in model 1. With further adjustment for sedentary activity, income-to-poverty ratio, education, and smoking, patients in quartile 4 of handgrip strength had 0.51 odds of poor glycaemic control (95% CI: 0.27–0.99). However, the reported association above vanished when further adjusted for insulin use (OR = 0.67; 95% CI: 0.35–1.28). In conclusion, findings may indicate an association between glycaemic control and muscle strength. This association may be altered by insulin use; further investigations are required.

## 1. Introduction

Diabetes has become one of the most ubiquitous health issues that the world is facing due to its global prevalence [1]. Diabetes mellitus is a metabolic health disease, characterised by elevated blood sugar levels due to genetics, acquired deficiency, or malfunction of insulin [2,3]. The number of diagnosed individuals with diabetes around the world is rapidly growing every year, increasing by 314 million cases within the 34 years from 1980 to 2014 and reaching 415 million in 2014, and the situation is getting worse [4]. Over 3 million people die worldwide every year due to diabetes and associated complications [5]. Diabetes is the seventh leading cause of death in the United States of America, where 69,091 deaths are due to diabetes and where diabetes has contributed to a further 234,051 deaths [6]. 

Several life-threatening complications are associated with diabetes [7], and several adverse health outcomes that develop gradually are associated with diabetes, including neuropathy, skin complications, eye complications, diabetic ketoacidosis, gastroparesis, and macrovascular diseases [8]. Uncontrolled diabetes not only affects patients’ quality of life but also greatly increases the health-care costs of a country. Data from the National Health and Nutritional Examination Survey (NHANES) have revealed that the average lifetime medical costs for a person with diabetes is as much as $85,200 with 53% of the costs used to manage complications [9]. However, one key assumption discussed in previous research is that the overall complications can be prevented via sustainable blood sugar control. 

Sedentary activity and muscle mass may be associated with good glycaemic control among patients with diabetes [10]. Previous studies showed that physical activity and resistance training had a positive impact on glycaemic control and muscle strength among patients with diabetes [11,12]. High-intensity resistance training can be used as a treatment method for type 2 diabetes and sarcopenia [11]. Several studies have investigated the association between muscle strength and risk of diabetes [13,14,15]. It is important to note that lower muscle strength that is positively correlated to muscle mass might not only be a cause of type 2 diabetes but also a consequence [16]. Findings on patients with type 2 diabetes indicate a significantly weaker muscle strength at both the lower (i.e., ankle and knee) and upper extremities (i.e., handgrip) when compared to age-matched controls, and especially in middle-aged and older adults [17]. The grip strength has typically been used as an important indicator of hand function and general muscle strength [18]. However, the underlying association between glycaemic control among patients with diabetes and grip muscle strength has not been addressed enough in the literature. The aim of the present cross-sectional study is to find whether there is an association between handgrip muscle strength and glycaemic control (i.e., Hb1Ac) among patients with diabetes.

## 2. Materials and Methods 

### 2.1. Subjects

**Figure 1:**
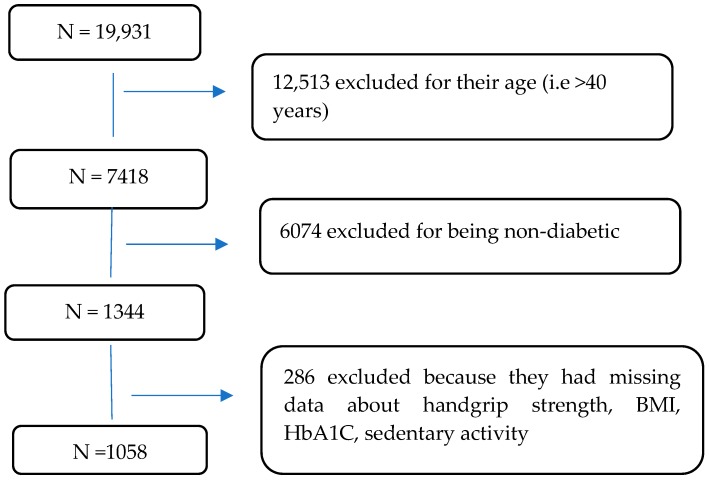
Flow chart of the study population.

NHANES recruits 5000 persons annually to measure the health and nutrition-related status of adults and children in the general US population. These surveys examine the standard activity of each individual and perform other health-related interviews. For laboratory analysis purposes, blood samples are withdrawn from each participant. Only participants aged 40 years and older attend both cohorts (2011–2012 and 2013–2014). The NHANES data on handgrip strength, BMI, HbA1c, and sedentary activity for individuals with known diabetes were included in the current study. The participants included in the study are self-reported with known diabetes, including both type 1 and 2 diabetes. Other participants with missing information on any of the previously mentioned variables and pregnant women were excluded. The total number of participants was 19,931. After excluding participants who did not meet the criteria or whose data were missing, the final sample size was 1058 participants (Figure 1). 

### 2.2. Dependent Variable 

#### Glycaemic Control 

The dependent variable for this study was glycaemic control among patients with diabetes, and HbA1c was used as the indicator of glycaemic control. HbA1c is the glycated haemoglobin formed by the exposure of haemoglobin to plasma glucose via non-enzymatic pathways. It is used as a marker for hyperglycaemia and to monitor blood plasma glucose levels over a prolonged period (2–3 months). As a biomarker of insulin sensitivity, it is used to identify patients with insulin resistance or in a prediabetic state and are at an elevated risk of developing diabetes [2]. HbA1c increases in response to a diet high in fat, smoking [19,20], and body fat in patients with diabetes [21]. HbA1c above 7% was considered to indicate poor glycaemic control [22].

### 2.3. Independent Variables

#### Muscle Strength 

Muscle strength refers to the ability of a muscle to exert a maximal force against a resistance in a single contraction. Adequate levels of muscular strength are needed for functional independence so that individuals can perform activities of daily living, such as moving, pushing, pulling, and lifting [23].

The handgrip test was performed using a calibrated handgrip dynamometer to measure muscle strength. Only participants with no prior conditions who could affect their grip strength were included in the sample. Prior to the measurements, the procedures were explained and demonstrated, and the grip size of the dynamometer was adjusted according to each participant’s hand size. Participants were instructed to squeeze the handle of the dynamometer as forcefully as possible while exhaling to prevent the rise of intrathoracic pressure. The arm was maintained straight and slightly away from the body. The measurements were taken in a standing position with the feet hip-width apart and toes pointing forward. After an initial hands and fingers’ warm-up and a practice trial, each participant was randomly assigned to start the test either with the left or right hand. Then, the same procedure was done for the other hand. The procedure tested each hand three times, with hands alternating between the trial. There was a 60-second rest between each trial for the same hand. The current study used combined grip strength, which is the addition of the largest reading from each hand and is expressed in kilograms [24].

### 2.4. Co-variates

#### 2.4.1. Height and Body Weight 

Height and body weight were measured using standardised procedures [25]. A stadiometer was used to measure participants’ standing height. It has a flexible head piece for adjustment purposes and a fixed vertical backboard. A standard digital scale was used to measure the weight of participants. While taking the weight measurements, the participants were asked to wear only underpants and the examination gown; the latter has a standard weight and is disposable. After recording both measurements (height and weight) for each participant, the BMI was calculated as kilograms of body weight divided by meters of height squared (kg/m^2^). 

#### 2.4.2. Sedentary Activity

The assessment of sedentary behaviour was done by interviewing the participants. They were asked to report the minutes of sedentary activity spent in a typical day. This typically includes sitting on a desk, traveling in a car or bus, reading, playing cards, watching television, or using a computer. The time spent sleeping was not counted as a sedentary activity. 

#### 2.4.3. Use of Insulin

The use of insulin was self-reported in the questionnaire. Participants were asked to answer with either ‘yes’ if they used insulin or ‘no’ otherwise. 

#### 2.4.4. Smoking Status 

Adults aged 18 years or older were asked about smoking, specifically cigarette use. The skilled interviewer used the CAPI system (computer-assisted personal interview system) in the participant’s home. 

#### 2.4.5. Alcohol Use

The alcohol questionnaire (ALQ) was administered at a mobile examination centre. For analyses, data are reported as a ‘yes’ or ‘no’ for drinkers and abstainers, respectively. For adults 18 years and older, questions were asked by trained interviewers using the CAPI system [24].

#### 2.4.6. Participant Demographics 

Demographic data about participants (family and individuals) were obtained through questions about gender, age, race, education, income, and other related information. Trained interviewers interviewed the participants at home with the aid of the CAPI system. The participants could choose to do the interview in either English or Spanish, or to have an interpreter present. Some questions were answered with the aid of hand cards, which allowed the user to choose the most accurate response from the options given. In some cases, the interviewer had to assist the interviewee by reading aloud the choices on the hand cards to make the selection easier [24].

#### 2.4.7. Income-to-poverty Ratio 

The federal income-to-poverty ratio was calculated using family income and the US Department of Health and Human Services’ poverty guidelines specific to the survey year.

### 2.5. Statistical Analysis 

Descriptive analysis was performed where means (standard deviations) were used to describe the continuous variables, and frequencies were used to describe categorical variables. ANOVAs or chi-square tests were used to test the differences between groups for continuous or categorical variables. Handgrip strength was presented as age- and gender-specific quartiles [26]. Three logistic regression models were used to investigate the association between poor glycaemic control and handgrip. The first model was adjusted for age, gender, and race; the second was further adjusted for sedentary activity, income-to-poverty ratio, education, smoking, alcohol drinking, protein intake, and BMI, and the third was further adjusted for insulin use. Statistical analysis was done using StataIC 15. 

## 3. Results

A total of 1058 participants were selected for the present study, which accounted for 5% of the total panel of participants. The participants’ characteristics are shown in Table 1, by quartiles of handgrip strength. The first quartile (Q1) has the least handgrip strength with 45.5 ± 13.4 kg; the second quartile (Q2) has 58.3 ± 14.1 kg. The third quartile (Q3) has 67.7 ± 15.9 kg, and the fourth quartile (Q4) has the highest handgrip strength mean value at 81.8 ± 20.0 kg. Of the total number of participants, 51% were males (*N* = 549), and 49% were females (*N* = 518). Gender was not associated with handgrip strength as the *p*-value was 1.00. The table also shows that older participants had less handgrip strength compared to younger participants. Q1 (65.6 years) had the least handgrip strength, and it keeps increasing until it reaches Q4 (60.2 years). The *p*-value was found to be < 0.001, which means that the association between handgrip strength and age is statistically significant. The table also describes the selected participants in terms of race (*p*-value < 0.001), education (*p*-value < 0.001), alcohol consumption (*p*-value < 0.001), BMI (*p*-value < 0.001), income-to-poverty ratio (*p*-value < 0.001), glycaemic control (*p*-value = 0.040), and use of insulin (*p*-value = 0.006); they all had significant differences by handgrip strength and statistically significant *p*-values. 

Logistic regression analysis was performed to further describe the association between the four quartiles of handgrip strength and poor glycaemic control (Table 2). Table 2 shows that there was no association between handgrip strength and glycaemic control after adjusting for age, gender, and race. However, there was an association across handgrip strength and glycaemic control after further adjusting for sedentary activity, income-to-poverty ratio, education, smoking, alcohol drinking, protein and energy intake, and BMI. Patients in Q4 of handgrip strength had 0.51 odds of poor glycaemic control (OR = 0.51; 95% CI: 0.27–0.99). Interestingly, this association disappeared when further adjustment to the model was done for insulin use. 

**Figure 2:**
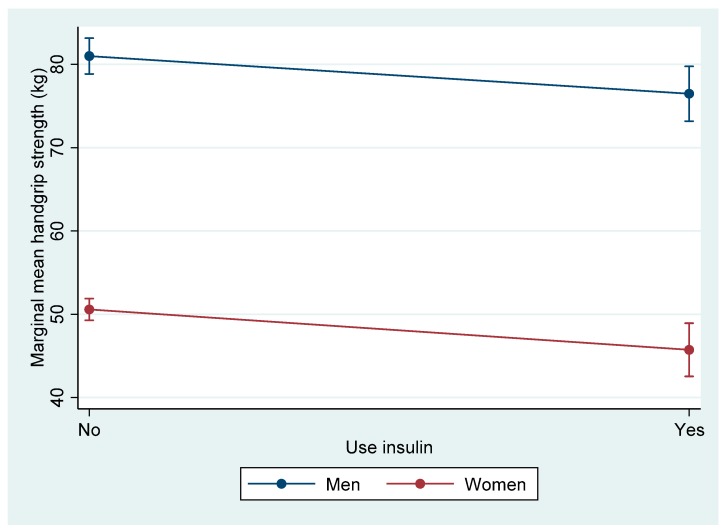
Marginal mean handgrip strength by insulin use in men and women. Note: Values were mean (95% CI), adjusted for age and race.

To understand the association between insulin use and muscle strength, the marginal mean of handgrip strength by insulin use in men and women was examined (Figure 2). Insulin users in both genders had less handgrip strength as compared to non-insulin users. *P*-values were 0.049 in men and < 0.001 in women when comparing the difference of handgrip strength by insulin use. There was no significant gender and insulin use interaction (*p* = 0.891).

## 4. Discussion

The present study aimed to better understand the association between muscle strength and glycaemic control among middle-aged and older patients with diabetes. Logistic regression analyses showed no significant association between grip strength and glycaemic control when accounting for differences in gender, age, and race. There was, however, an association (*p* = 0.073) when controlling for variations in biological (i.e., BMI), behavioural and dietary (i.e., sedentary activity, smoking, alcohol drinking, and protein and energy intake), and educational and socio-economic variables (i.e., education and income-to-poverty ratio). Interestingly, the association disappeared when the model was further adjusted for insulin use, suggesting a mediating role of insulin use.

The lack of statistical significance found in the association between grip strength and glycaemic control might seem counter-intuitive when compared to findings of other published studies. It is important to acknowledge that the present study’s population was homogeneous in a sense that all participants were patients with diabetes, while most literature studies have included both healthy individuals and patients with diabetes. For instance, comparative cross-sectional studies showed that diabetes or poor glycaemic control are associated with lower muscle strength [17,27,28], while many prospective studies found lower muscle strength to be associated with a higher incidence of type 2 diabetes or hyper-glyceamia over periods of 3, 7.5, and 10 years [15,29,30]. 

In a three-year follow-up on 1840 older adults, a greater decline in lower-limb strength and mass were also reported for older type 2 diabetes patients as compared to healthy adults of similar age [30]. However, authors showed that changes in arm strength, on the other hand, were not significantly different between the patients and healthy groups. The later might offer a further interpretation as to the lack of significant association found in this study, where grip strength was examined and considered representative of overall strength. Further studies could consider assessing the strength of larger muscle groups (i.e., legs) when investigating the muscle strength and glycaemic control association in patients with diabetes. Nevertheless, our first result seems to go in line with that of a study on 2318 participants that found no association between handgrip strength and risk of type 2 diabetes after a 10.7-year follow-up, due to lack of statistical power [31].

It is also important to mention that the lack of significant association between grip strength and glycaemic control was present after an initial adjustment to age, gender, and race. Indeed, patients in the highest-strength quartile were significantly younger than those in the lowest-strength quartile, which is in line with previous reports indicating a gradual decline in grip strength after the fourth decade [32,33]. However, the surprising lack of significant gender differences in grip strength [32,33] might be underlying a potential interaction between gender and age in the current population. 

The present study found a trend of association (*p* = 0.073) between muscle strength and glycaemic control after accounting for differences in sedentary activity, income-to-poverty ratio, education, smoking, alcohol drinking, and BMI. The lack of a strong association may also be explained by the participants’ glycaemic control that was not severely deteriorated enough to detect an association. Amongst the co-variates, results showed that patients in the highest-strength quartile had better glycaemic control as compared to those in the lowest quartile [27,29]. Insulin use in the current study confounded the results; the trend of association between muscle strength and glycaemic control vanished when insulin use was further controlled for. Those findings suggest that the impact of insulin use on muscle strength may be mediated by insulin resistance. Indeed, in a study on 959 participants, handgrip strength was inversely related to insulin resistance (*p* = 0.025) and fasting insulin levels (*p* = 0.017) [26]. Moreover, Brooks et al. (2007) showed that strength training improves muscle quality, glycaemic control, and insulin resistance in Hispanic older adults with type 2 diabetes [34]. Taken together, it is likely to assume that insulin use could theoretically and indirectly improve muscle strength. A further analysis reveals that both male and female insulin users had less grip strength as compared to non-insulin users (Figure 2). This later analysis might seem to contradict our previous interpretation; however, it can also be explained by the fact that patients in the lower-strength quartile were more likely to be insulin users as compared to the highest-strength quartile. The exact nature of the relationship cannot be ascertained at this stage from the present study. 

This study had some limitations. There was no information about inflammatory biomarkers, which are associated with the development of type 2 diabetes. Doses of insulin in each participant are unknown, and medication use is not included. Another limitation is that the study does not consider the duration of diabetes in participants, or the existence or severity of any neuropathy. Those factors were found to be important ones affecting muscle strength in patients with diabetes. Moreover, the present study only looked at sedentary behaviour as an adjustment factor, while physical activity was not considered. Indeed, given the dissociation between sedentary behaviour and physical activity, sedentary individuals can still meet the physical activity recommendations for health. It has been shown that ‘both normal people and patients with type 2 diabetes can have an improved glucose tolerance through continuous physical activity’. Therefore, results of the current study do not reflect any potential effect of participants’ actual physical activity levels, which would be beneficial to account for in future studies. 

## 5. Conclusions

In conclusion, handgrip strength is associated with glycaemic control in patients with type 2 diabetes. However, the mediating role of insulin use on the association between strength and glycaemic control in patients with diabetes would merit further investigation. 

## Figures and Tables

**Table 1:** Sample characteristics by quartiles of handgrip strength (*N* = 1058).

	Q1	Q2	Q3	Q4	*p*-Value
	*N* = 268	*N* = 263	*N* = 266	*N* = 261	
Handgrip strength (kg)	45.5 (13.4)	58.3 (14.1)	67.7 (15.9)	81.8 (20.0)	< 0.001
Age (years)	65.6 (12.0)	62.8 (11.4)	62.2 (10.5)	60.2 (9.8)	< 0.001
Gender					1.00
Men	138 (51.5%)	133 (50.6%)	135 (50.8%)	134 (51.3%)	
Women	130 (48.5%)	130 (49.4%)	131 (49.2%)	127 (48.7%)	
Race					< 0.001
NH White	103 (38.4%)	95 (36.1%)	95 (35.7%)	86 (33.0%)	
NH Black	57 (21.3%)	49 (18.6%)	84 (31.6%)	127 (48.7%)	
Mex. American	41 (15.3%)	43 (16.3%)	38 (14.3%)	20 (7.7%)	
Other race/ethnic	67 (25.0%)	76 (28.9%)	49 (18.4%)	28 (10.7%)	
Education					< 0.001
< 11 grade	117 (43.8%)	90 (34.2%)	83 (31.3%)	53 (20.3%)	
HS dipl. or GED	58 (21.7%)	51 (19.4%)	72 (27.2%)	62 (23.8%)	
Some college	53 (19.9%)	75 (28.5%)	70 (26.4%)	93 (35.6%)	
> college	39 (14.6%)	47 (17.9%)	40 (15.1%)	53 (20.3%)	
Smoking					0.82
Never	137 (51.1%)	126 (47.9%)	133 (50.0%)	130 (49.8%)	
Former	92 (34.3%)	95 (36.1%)	87 (32.7%)	97 (37.2%)	
Current smoker	39 (14.6%)	42 (16.0%)	46 (17.3%)	34 (13.0%)	
Alcohol drinking					< 0.001
No	85 (31.7%)	71 (27.0%)	77 (28.9%)	73 (28.0%)	
Yes	102 (38.1%)	132 (50.2%)	141 (53.0%)	145 (55.6%)	
Missing	81 (30.2%)	60 (22.8%)	48 (18.0%)	43 (16.5%)	
BMI (kg/m^2^)	30.7 (7.0)	31.5 (7.3)	32.7 (7.7)	33.8 (6.8)	< 0.001
Sedentary activity					0.21
< 3 hrs	41 (15.3%)	58 (22.1%)	47 (17.7%)	51 (19.5%)	
3–6 hrs	77 (28.7%)	89 (33.8%)	85 (32.0%)	79 (30.3%)	
6+ hrs	150 (56.0%)	116 (44.1%)	134 (50.4%)	131 (50.2%)	
Income-to-poverty ratio					< 0.001
< 1.30	119 (47.6%)	105 (43.6%)	97 (39.8%)	59 (24.1%)	
1.3–3.5	83 (33.2%)	96 (39.8%)	92 (37.7%)	104 (42.4%)	
> 3.5	48 (19.2%)	40 (16.6%)	55 (22.5%)	82 (33.5%)	
HbA1c (%)	7.5 (2.0)	7.4 (1.7)	7.6 (1.9)	7.2 (1.7)	0.078
Poor glycaemic control	130 (48.5%)	132 (50.2%)	141 (53.0%)	107 (41.0%)	0.040
Use of insulin	94 (35.1%)	75 (28.5%)	68 (25.6%)	57 (21.8%)	0.006

Note: Data are presented as mean (SD) for continuous measures and N (%) for categorical measures.

**Table 2:** Association between quartiles of handgrip strength and poor glycaemic control (*n* = 1058).

	Q1	Q2	Q3	Q4	*p* for Trend
Model 1	1.00	0.99 (0.61-1.62)	1.34 (0.76-2.34)	0.73 (0.44-1.23)	0.434
Model 2	1.00	0.86 (0.45-1.62)	1.07 (0.57-1.99)	0.51 (0.27-0.99)	0.073
Model 3	1.00	1.09 (0.59-2.03)	1.37 (0.70-2.69)	0.67 (0.35-1.28)	0.319

Note: Weighted sample size is 17,605,974. Model 1 adjusted for age, gender, and race. Model 2 further adjusted for sedentary activity, income-to-poverty ratio, education, smoking, alcohol drinking, protein and energy intake, and BMI. Model 3 further adjusted for insulin use.
